# Low Cost Inkjet Printed Smart Bandage for Wireless Monitoring of Chronic Wounds

**DOI:** 10.1038/srep28949

**Published:** 2016-06-29

**Authors:** Muhammad Fahad Farooqui, Atif Shamim

**Affiliations:** 1 Electrical Engineering Program, 4700 King Abdullah University of Science and Technology (KAUST), Thuwal 23955-6900, Saudi Arabia

## Abstract

Chronic wounds affect millions of patients around the world and their treatment is challenging as the early signs indicating their development are subtle. In addition, a type of chronic wound, known as pressure ulcer, develops in patients with limited mobility. Infection and frequent bleeding are indicators of chronic wound development. In this article, we present an unprecedented low cost continuous wireless monitoring system, realized through inkjet printing on a standard bandage, which can send early warnings for the parameters like irregular bleeding, variations in pH levels and external pressure at wound site. In addition to the early warnings, this smart bandage concept can provide long term wound progression data to the health care providers. The smart bandage comprises a disposable part which has the inkjet printed sensors and a reusable part constituting the wireless electronics. This work is an important step towards futuristic wearable sensors for remote health care applications.

Advances in wearable and flexible electronics along with the growth of wireless networking have created new paradigms of applications for smart living. One important application is the development of advanced health monitoring systems that enable people to stay better informed about physiological changes in their bodies. Such systems could play a key role in developing a healthy environment for better living and increasing the quality of life. Increase in population and rising cost of healthcare services have also created a growing demand to monitor a patient’s health in personal environment outside of a hospital. Therefore, wearable sensors are being developed to monitor various physiological parameters of the human body. A number of sensors have been developed to monitor the vital signs of the human body including body temperature^1^, heart rate^2^, electrocardiogram^2^,^3^ and blood pressure^4^. A few sensors have also been developed to monitor the brain activity by recording electroencephalogram^5^.

Chronic wound monitoring is one area of human health that has received relatively less attention from the research community. Chronic wounds present a significant challenge to modern health care providers as they affect more than 9 million people in the United States and Europe. The annual costs to treat chronic wounds exceed US$ 39 billion^6^,^7^. Wounds can be broadly classified into two categories, acute wounds and chronic wounds. Acute wounds follow an orderly healing process and close in a short period of time whereas chronic wounds do not follow an orderly healing process in a predictable amount of time. Chronic wounds either do not heal or heal very slowly and reoccur after healing. Wounds that do not heal within three months are termed chronic^8^. Usually, diabetic, obese and elderly people tend to suffer more from them^9^,^10^ and if the treatment is not done in a timely manner, infections and complications can occur in these wounds. Diagnosis and the treatment of chronic wounds are quite complex and pose a major challenge to the health care staff. The reason is that the initial symptoms of a chronic wound are very subtle and it is hard to differentiate them from those of an acute wound^11^. It is therefore difficult to assess whether the healing process has been perturbed or not. One of the major causes of disruption of the healing process is bacterial infection. An infection can result in the overgrowth of a newly formed capillary rich granulation tissue over the wound. This condition is termed as overgranulation and it can hamper the healing process. Overgranulation results in a protruding, friable flesh that is very sensitive and bleeds easily. As such, chronic wounds often show frequent and irregular bleeding^12^. Changes in pH values have also been related to the presence of infection. An infected wound shows slightly basic pH due to certain enzyme activities, bacterial colonization and formation of protein structures^13^.

Chronic wounds can be classified into three major categories, namely diabetic foot ulcers, venous leg ulcers and pressure ulcers. These wounds vary in dimension, depth and the amount and composition of wound exudate. Pressure ulcers can develop if a part of the body is under sustained pressure for a longer period of time^14^. Such a situation usually occurs when a patient lies in the same position for a long time after any surgical operation in the intensive care unit (ICU) or if the patient has limited mobility. Obesity and inactivity due to age can increase the probability of pressure ulcers. The pressure causes reduced blood flow in the tissue that can lead to tissue death, and subsequently, an infection.

There is currently no commercially available wireless device to continuously monitor the wound healing process and patients rely on medical staff for physical inspection of the wound, which requires repeated trips to clinics or prolonged hospitalization. After surgical treatments, patients are manually repositioned every hour to relieve pressure in order to avoid the development of pressure ulcer. Few devices have been reported in the literature that monitor parameters related to the wound healing process. These include a bandage in solution form that can be painted onto the skin to form a thin film^15^. The film emits oxygen dependent phosphorescence that can be used to map the oxygen levels of the underlying skin tissue. A polyaniline (PANI) based potentiometric sensor realized on a bandage strip has been reported to detect the pH levels of the wound^16^. In another example, a flexible electrode array has been developed through the inkjet printing of gold nanoparticle on flexible polyethylene naphthalate to measure the impedance spectrum of the tissues for early detection of pressure ulcers^17^. Electrodes have been demonstrated to measure moisture levels^18^ as well as bacteria^19^ in wound dressings. A hydrogel based wireless sensor for pH monitoring of wounds has been fabricated^20^. These reports describe sensing of a single parameter related to the wound healing process. Also, the designs are not optimized for wearability and cost and some also require complex fabrication processes. None of them has an integrated wireless module, thus rendering them unsuitable for remote health monitoring applications. A close example is a wireless telemetry system that has been demonstrated to monitor the pressure and humidity levels in compression bandages^21^. However, the system dimensions are large as commercial sensors are used that are not integrated and are connected through wires.

Wound healing is a complex process, therefore an accurate diagnosis and treatment may require information about a number of factors that can affect wound healing. An attractive solution would be a low cost, wearable, compact, wireless, real-time wound monitoring system that can be worn by patients in everyday life. Such a system should be able to issue early warnings to the patients regarding any abnormality in the healing process, as well as wirelessly send the data recordings of multiple parameters related to the wound healing process to the remote medical staff. In this way, health care providers would have access to the history of wound progression that could be vital in the diagnosis and treatment process. Adhesive bandages are most commonly used to protect wounds from the external environment and augment rapid healing. Here, we have developed for the first time, a complete wearable system to wirelessly monitor chronic wounds using a simple bandage strip. The system, termed as smart bandage, comprises inkjet printed sensors that have been realized on a disposable bandage to monitor bleeding, pH levels and external pressure on the wound site. Moreover, sensor and wireless electronics have been smartly integrated on the bandage that can be detached and reused on another bandage, thus maintain the disposability of the bandage strip in contact with the wound. The wearable smart bandage can alert the patient and the health care providers regarding any abnormality in the wound healing process through the integrated wireless module. Continuous monitoring also facilitates acquisition of long term wound progression data. The sensor as well the circuit board and antenna have been developed using low cost inkjet printing process and flexible substrates which make it attractive in terms of wearability and cost. The smart bandage system can be used to monitor any type of chronic wound regardless of its size as the sensor dimensions are scalable. Here, just to show a proof of concept, a commercial bandage strip has been used to implement the system. Our smart bandage can be worn in daily life and provides an attractive solution for remote health monitoring, and thus reduces the burden on health care services to meet the growing demands of the population.

## Results

### System Design and Operation

**Figure 1:**
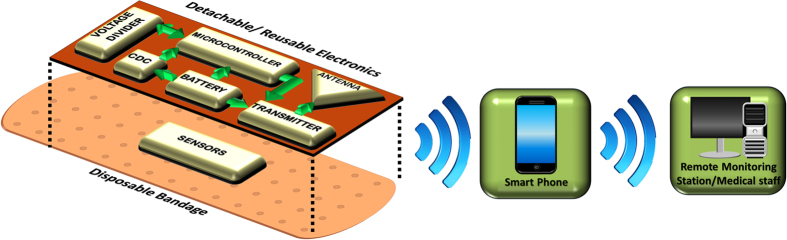
System Design. The smart bandage assembly comprises two parts: a disposable part and a reusable part. The sensors are printed on a disposable bandage strip that can be disposed off after use. The wireless electronics which include inkjet printed circuit board and antenna are made on a kapton tape that can be detached and reused on another bandage. This approach reduces the cost of the system and maintains disposability of the bandage which has been in contact with the wound. The bandage can communicate wirelessly to a patient’s smart phone which can then connect to remote health care providers over the mobile network or the internet.

The system level design is depicted in Fig. 1. As mentioned above, the system has been designed as a combination of a disposable part and a reusable part to reduce the cost. The sensors to detect bleeding, pH levels and external pressure on the wound are realized on a disposable bandage whereas the electronics are integrated on a flexible kapton tape which can be detached and reused multiple times. Two types of sensing mechanisms are used. A capacitive sensor detects bleeding as well as pressure levels on the wound. A resistive sensor detects the pH levels on the wound. The changes in capacitance and resistance are processed by the electronics and the information is sent in a wireless fashion using IEEE 802.15.4 standard that operates around 2.4 GHz. The detachable electronics comprise a transmitter with an embedded microcontroller, a capacitance to digital converter (CDC), an LED to inform the patient about the status of the bandage and a battery to power the system. The wireless communication is done through an inkjet printed loop antenna that is integrated with the circuit. The smart bandage can wirelessly communicate with a personal smart phone to provide wound progression data in a patient’s personal environment. This data can then be sent from the patient’s smart phone to remote health care providers using either the mobile network or the internet.

The CDC continuously compares the sensor capacitance to a reference capacitance. When the sensor capacitance becomes greater than the reference capacitance as a result of bleeding or external pressure, the CDC outputs logic high. This output goes into one of the ports of the microcontroller. The output from the resistive sensor is processed in a similar fashion to the capacitive sensor. The change in resistance of the sensor is converted into voltage change which goes into another port of the microcontroller. The microcontroller continuously monitors the voltage level on these two ports and as soon as it detects a signal, it activates the transmitter as well as the LED.

### Sensor Design

**Figure 2:**
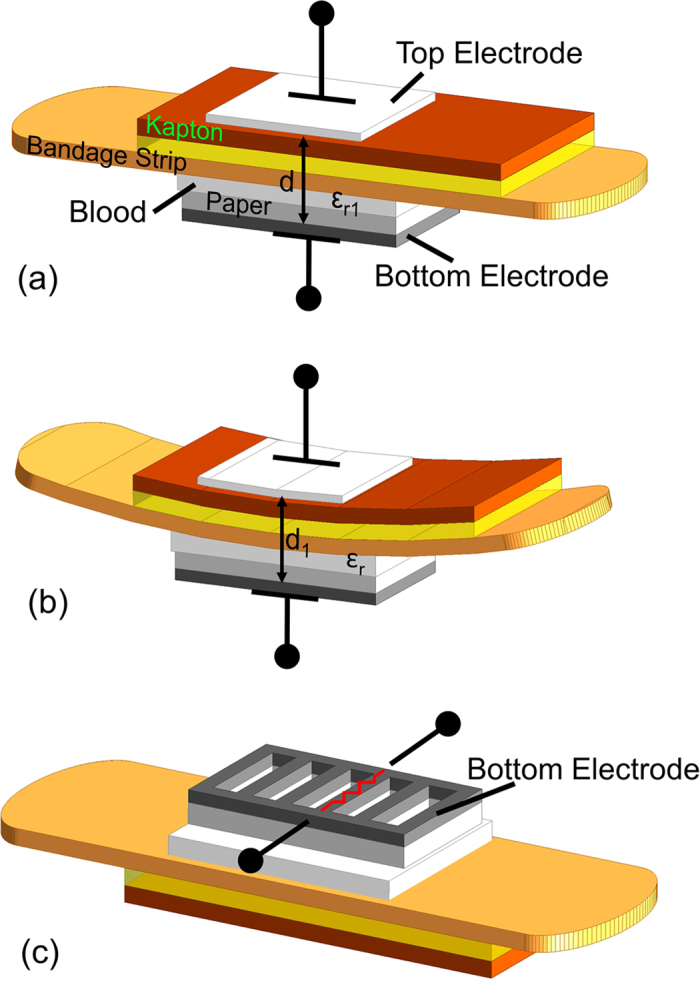
Sensor designs. Two sensor electrodes are mounted on the top and the bottom side of the bandage forming a capacitor. (**a**) The bleeding is sensed when the blood from the wound penetrates into the bandage and changes the dielectric constant to *ε*_r1_ which is greater than *ε*_r_. (**b**) The external pressure is sensed when the pressure changes the distance between the electrode to *d*_1_ which is less than *d*. (**c**) The pH levels are sensed when the resistance of the carbon based bottom electrode changes in response to pH.

As mentioned before, the smart bandage senses three parameters related to chronic wounds which are irregular bleeding, pH levels and external pressure. In order to sense these parameters, two types of sensing mechanisms are used namely capacitive sensing and resistive sensing. These mechanisms are highlighted in Fig. 2. A capacitor is formed by placing two electrodes on either sides of the bandage strip. The capacitance, *C*, of a metal-insulator-metal capacitor is given by *C* = *ε*_0_*ε*_r_ *A*/*d*, where *A* is the area of the electrodes, *d* is the separation between the electrodes, *ε*_r_ is the dielectric constant of the insulator between the electrodes and *ε*_0_ is the permittivity of free space. The bleeding is sensed due to the change in *ε*_r_ of the capacitor. In the case that the wound starts to bleed, blood from the wound will penetrate into the bandage and change the *ε*_r_ of the bandage resulting in a change of capacitance. The external pressure is sensed due to the change in *d*. When pressure is applied to the bandage, the two electrodes of the capacitor are pressed together and result in a smaller *d*. The change in *d* causes a change in capacitance. The pH levels on the wound site are detected by changes in resistance of one of the electrodes (carbon based) placed on the bandage.

**Figure 3:**
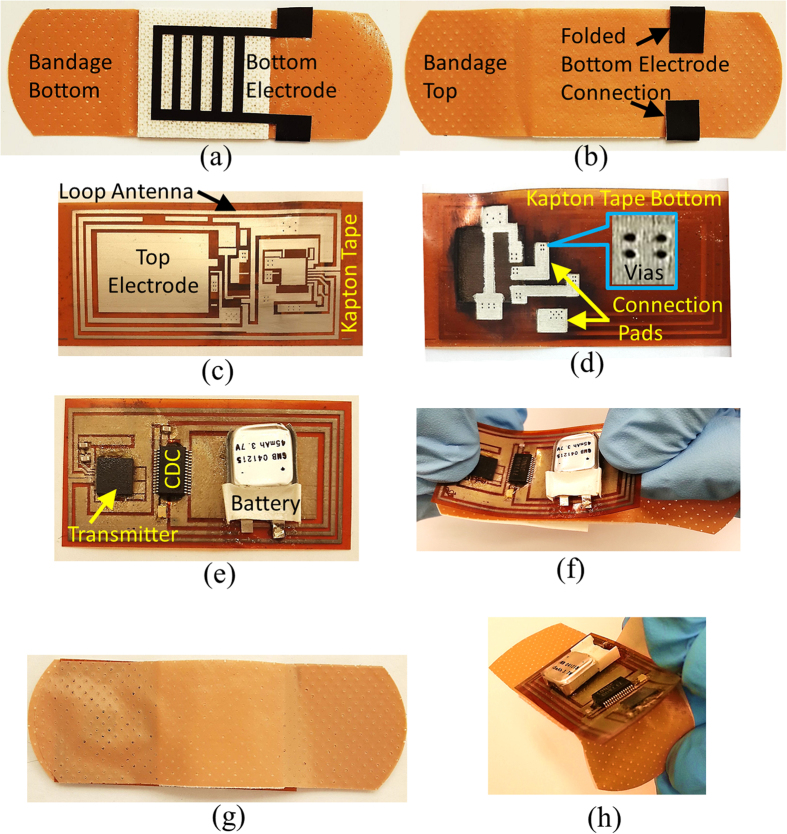
Fabrication and assembly steps. (**a**) Bottom side of the bandage showing printed carbon based sensor electrode. (**b**) Top side of the bandage showing folded connection of the bottom electrode. (**c**) Top side of inkjet printed circuit board on kapton tape. (**d**) Bottom side of the circuit board highlighting vias and pads for connecting bottom sensor electrode. (**e**) Detachable electronics comprising Kapton printed circuit tape after mounting of the components. (**f**) Electronics being mounted on the bandage. (**g**) Smart bandage enclosed by a bandage strip acting as a cover package. (**h**) The detachable electronics can be easily removed from the bandage after use.

The two electrodes are placed on the top and bottom side of the bandage. The bottom electrode has a rectangle mesh geometry that has lines of width 1 mm and gaps of 2 mm as shown in Fig. 3a. This geometry allows the blood to seep into bandage and also allows the bandage pad to be in contact with the wound. In order to connect this bottom electrode to the electronics at the top side of the bandage, part of this electrode has been folded across the bandage so that it is accessible from the top as shown in Fig. 3b. The top electrode is in the form of a rectangular patch with dimensions of 14 mm × 20 mm, and is part of the detachable electronics as shown in Fig. 3c. The two electrodes are therefore separated by the thickness of the bandage strip. The bottom electrode also forms the resistive sensor in addition to being a part of the capacitive sensor. Since the bandage strip has a limited area, this technique efficiently utilizes the area on the bandage by integrating two sensing mechanisms in one set of electrodes. The bottom electrode, as shown in Fig. 3a, is made using carbon based ink that is compatible with screen printing process. When exposed to a solution containing hydronium ions (H_3_O^+^) in the case of an acid or hydroxide ions (OH^−^) in the case of a base, the carbon reacts with these ions which results in a change of its conductivity.

### Antenna Design

A planar rectangular loop antenna has been designed for this application. A loop antenna has been chosen because of its planar design, differential operation and efficient area utilization. As shown in Fig. 3c, the area inside of the loop can be utilized to place the electronics resulting in a compact design, since there is limited area on the bandage to place the sensor as well as the electronics. The loop antenna has been simulated in the presence of electronics located inside the loop to include the effect of the circuit on antenna performance. The simulations have been carried out in Ansys HFSS. The results show that the antenna radiates an omnidirectional pattern as expected and that the sensor electronics has little effect on the operation of the antenna. The antenna operates at around 2.4 GHz and has dimensions of 23.8 mm × 46.5 mm. The maximum simulated gain of the antenna is around 0.5 dBi.

### Printed Circuit Board on Kapton and System Integration

Conventional printed circuit boards (PCBs) are hard, bulky and rigid. The use of such PCBs for a wearable application is not suitable. In this work, a double-sided, detachable PCB has been inkjet printed on kapton adhesive tape to keep the design light weight, flexible, conformal and comfortable for wearability as shown in Fig. 3c,d. The circuit consists primarily of Texas instruments^®^ CC2530 transmitter chip and an ON Semiconductor LC717A00AJ CDC chip with very few external components, which include an LED, a crystal resonator and a battery as shown in Fig. 3e. The transmitter includes an on-chip microcontroller to store the bleeding, pH and pressure data of the wound and can support data rates of up to 250 kbps which is well above the requirement for this particular application. The circuit is powered by a thin lithium-polymer battery with dimensions of 2 mm × 12 mm × 12.5 mm. The thin (2 mm thick) battery from PowerStream Technology, is light weight (0.45 gm.) and suitable for this design. If the bandage communicates after every 5 minutes in order to provide wound progression data, the battery can operate the system for up to 55 hours. The battery can also be replaced or recharged using commercially available charging system by connecting them to the bandage. For this purpose, appropriate connections can be provided on the detachable electronics portion of the bandage. As mentioned above, the complete circuit is located inside the loop antenna to optimize the space. A differential feed line connects the transmitter IC to the antenna. The kapton PCB tape is shown in Fig. 3e after mounting of the components. The circuit makes electrical contact with the bandage through pads placed on the bottom side of the kapton PCB tape. When the kapton tape is attached, these pads connect to the folded portions of the bottom electrode on the bandage, which can be seen in Fig. 3b. The use of inkjet printing to realize circuit board on Kapton tape makes the electronics detachable and reusable. The tape can be attached to the bandage before it is worn by the patient as highlighted in Fig. 3f. Finally, for simplicity of the proof of concept, the detachable electronics has been covered from the top by another disposable bandage strip to demonstrate packaged smart bandage as shown in Fig. 3g. Once used, the disposable bandage strip can be removed, as shown in Fig. 3h, and the electronics can be reused on another bandage, which reduces the overall cost of the system.

### Smart Bandage Fabrication

**Figure 4:**
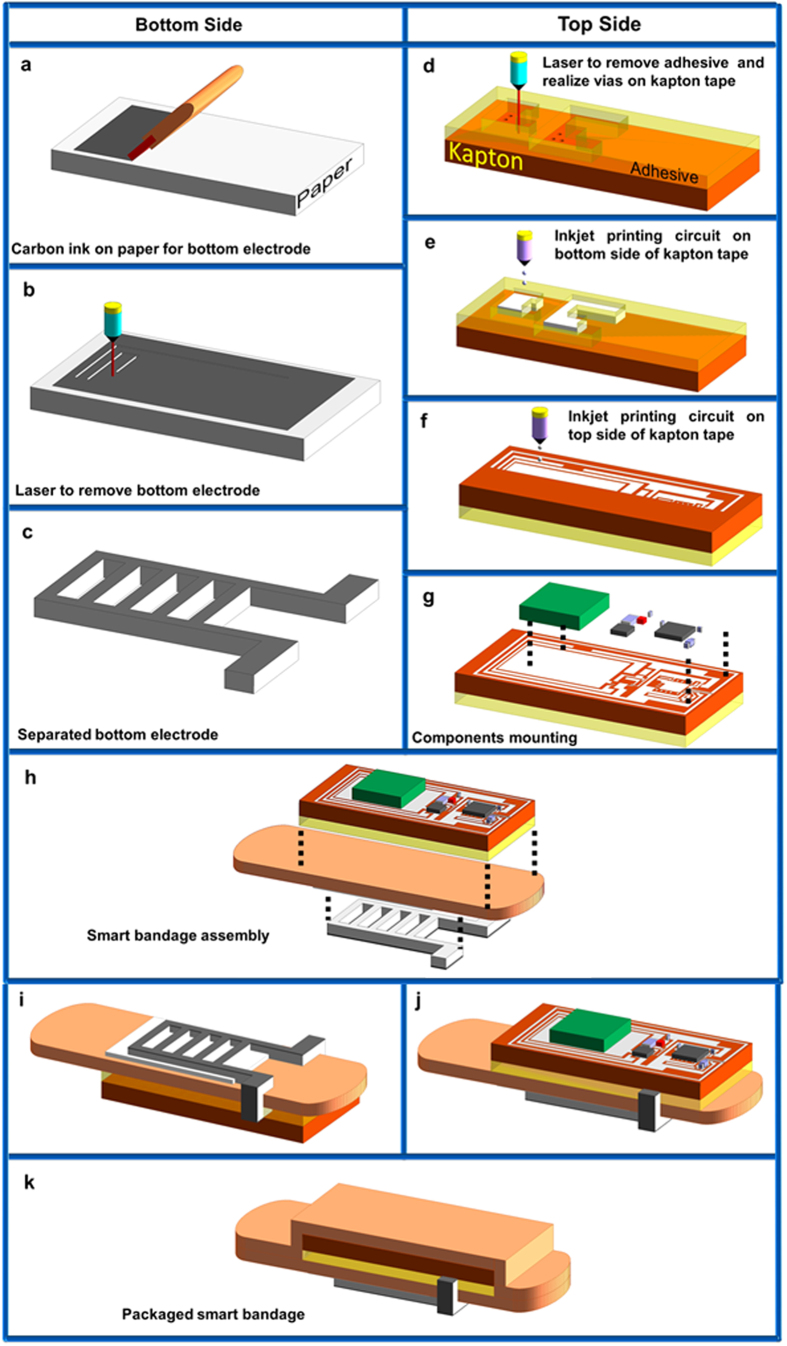
Fabrication process. Two parallel fabrication steps are involved for the realization of the smart bandage system. Bottom electrode fabrication using carbon ink on paper and wireless electronics fabrication using double-sided inkjet printed circuit board on kapton. (**a**) Carbon ink spreading on paper. (**b**) Cutting of bottom electrode using laser. (**c**) Separated bottom sensor electrode. (**d**) Processing of bottom side of the kapton tape. (**e**) Inkjet printing bottom side of the circuit board. (**f**) Inkjet printing top side of the circuit board. (**g**) Mounting of circuit components using silver epoxy. (**h**) The bottom electrode and the integrated electronics are mounted on the disposable bandage strip. (**i**) Bottom view of the smart bandage. (**j**) Top view of the smart bandage. (**k**) Finally another disposable bandage strip is put on top of the detachable electronics for packaging purpose.

The bandage is fabricated using low cost inkjet printing process on flexible substrates. The fabrication of the bandage takes place in two parallel processes. The steps involved in these two processes are highlighted in Fig. 4. The first process involves the fabrication of the bottom sensor electrode on paper substrate. Paper is one of the cheapest material and costs around one tenth of plastic^22^. It is flexible, biocompatible and biodegradable, thus it is extremely suitable for this application. Carbon ink is used for the metallization of bottom electrode. The second process involves the fabrication of sensor electronics as well as the top sensor electrode on kapton tape, which is placed on the top of the bandage. Silver nanoparticle ink is used for the inkjet printing of top electrode, circuit board and antenna. After mounting of the components, the detachable sensor electronics as well as the bottom sensor electrode are attached to a disposable bandage strip. A commercially available adhesive bandage strip is used in this work which has a length of 3 in. and a width of 1 in.

### Bleeding and Pressure Sensing

**Figure 5:**
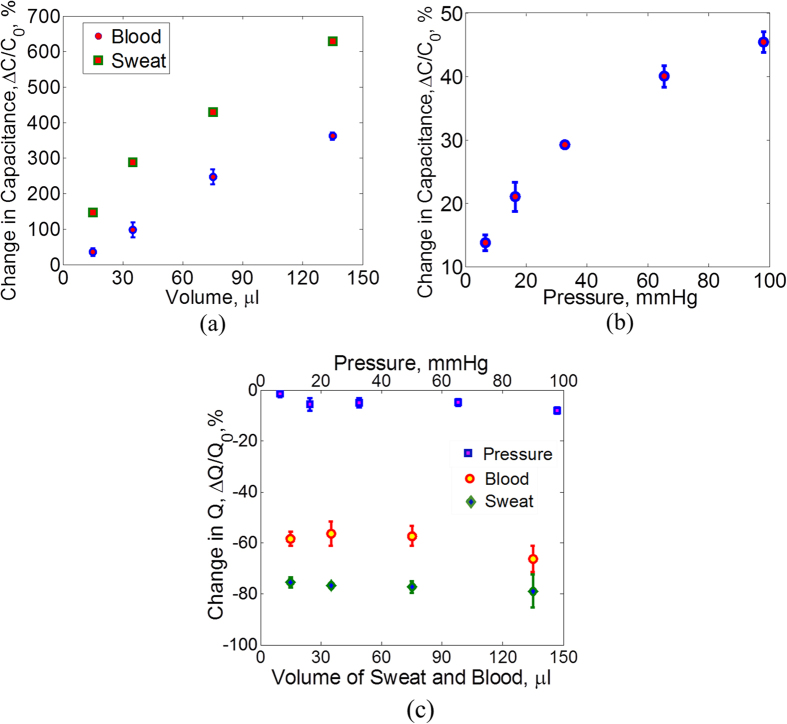
Capacitive sensor characterization. (**a**) Sensor capacitance is measured for fixed volumes of sweat and blood (where sweat and blood are represented by equivalent properties fluids). The difference in capacitance change for blood and sweat is significant which shows that the senor will be able to detect blood even in the presence of sweat. The error bars show the variation in change of capacitance for different samples. (**b**) Sensor capacitance is also measured under the influence of external pressure on the bandage in the range of 5–100 mmHg. The maximum error in the measurements is around ±2.3%. (**c**) The change in Q is different for the cases of external pressure, bleeding and sweating. This difference allows the selective detection of the three stimuli.

The capacitive sensor placed across the bandage senses bleeding. Blood has a dielectric constant (ε_r_) of 58 at normal body temperature^23^ and a loss tangent (tanδ) of 1.2^24^,^25^. In order to mimic blood, a mixture of ethanol and saline is made that has a similar ε_r_ and tanδ as that of blood (see Supplementary Fig. S1). Fixed volumes of this mixture have been put on the bandage and the capacitance is measured across the two sensing electrodes. The capacitance is proportional to the dielectric constant between the electrodes. As can be seen from Fig. 5a, a few micro liters of fluid have made a significant change in capacitance. The initial capacitance value, *C*_0_, of different samples varies from 2.4 pF to 2.7 pF which can be due to the slight misalignment of the top and bottom electrodes as they are placed manually. The variation in capacitance change as indicated by the errors bars can be due to the discrepancy in depositing the blood solution on the bandage. There is a possibility that small amount of solution stays on top of the bottom electrode which prevents it from penetrating into the bandage.

When a patient wears the bandage, other body fluids such as sweat and wound exudate can also seep into the bandage, which can result in an erroneous signal. An evaluation of capacitance change under influence of such body fluids has also been carried out. Sweat and exudate are mostly water and contain electrolytes^26^,^27^. Sweat has ε_r_ of 86 and tanδ of 1.08^28^. In order to mimic such fluids, a saline solution has been used (see Supplementary Fig. S1). Fixed volumes of saline solution are put on the bandage and the capacitance change is measured. As can be seen in Fig. 5a, there is a significant difference in the change of capacitance for saline and blood which can help in distinguishing the effect of sweat in the sensor readings.

In order to measure external pressure using the capacitive sensor, different values of mass are put on the bandage in an area, *A*, of around 1 cm^2^. The standard calibration weight set from Mettler Toledo is used for this purpose. Masses, *m*, of 50 gm, 100 gm, 150 gm and 200 gm and 300 gm are put on the bandage. The pressure, *P*, corresponding to each mass is calculated using the relation *P* = (*m * *  *g*)*/A* where *g* is the acceleration of gravity. The results are shown in Fig. 5b. A pressure of around 6 mmHg produces a capacitance change of around 13%. Studies have shown that a pressure of more than 60 mmHg can result in muscle damage if applied for more than an hour^29^,^30^. The maximum variation in the capacitance change under external pressure is around ±2.3%.

The quality factor, Q, of the capacitive sensor has also been measured for different values of external pressure as well as for different volumes of blood and sweat mimicking fluids in order to investigate the selectivity of the sensor for these three stimuli. The change in Q for applied pressure as well as blood and sweat volumes is shown in Fig. 5(c). It can be seen that in the case of external pressure, there is a little change in Q of the sensor (less than 8% decrease) for increasing values of pressure, whereas in the case of blood and sweat, there is a significant decrease in the Q which is expected due to the lossy nature of these fluids. Therefore, external pressure can be clearly distinguished from blood and sweat exposure for all values of pressure, bleeding and sweating by measuring the Q values of the sensor in addition to the capacitance.

Moreover, due to the difference in dielectric constant and loss tangent of blood and sweat, there is a noticeable difference in the change of Q for both of these fluids. In the case of blood, the mean decrease in Q varies between 56–66% for different volumes whereas for sweat, it varies between between 75–79%. Also, the decrease in Q is not affected much by the volume of the fluids. Hence, by measuring the Q of the sensor, it is also possible to isolate the readings of bleeding and sweating in addition to external pressure. However, it is to be noted that in order to measure Q, the capacitance to digital converter (CDC) in the attached electronics of the bandage needs to be replaced by an impedance to digital converter (IDC) which is also available commercially^31^.

### pH sensing

**Figure 6:**
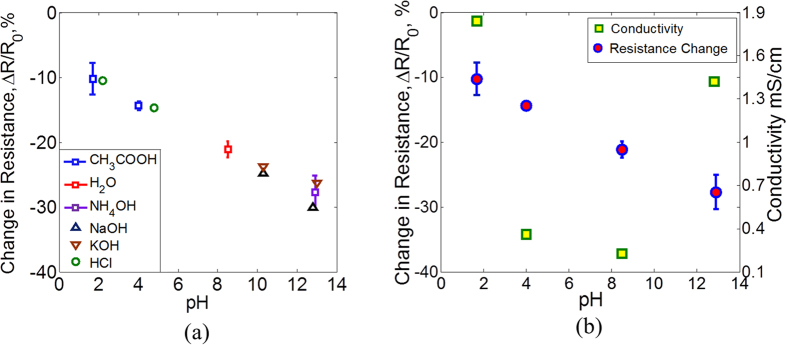
Resistive sensor characterization. (**a**) The resistance value decreases for increasing levels of pH. The maximum variation in the resistance change is around ±2.6% as shown by the error bars for CH_3_COOH, H_2_O and NH_4_OH. The variation is small for the range of pH values in chronic wounds (pH 5.4 to 8.6). Also different acids and bases with same pH produce similar resistance change. (**b**) Conductivities of the solutions show different trend against pH as compared to resistance change.

For pH measurements, different acid and base solutions have been used in addition to water which is close to neutral pH. For acidic pH, acetic acid (CH_3_COOH) and hydrochloric acid (HCl) have been used and for base, ammonium hydroxide (NH_4_OH), potassium hydroxide (KOH) and sodium hydroxide (NaOH) have been used. Acidic solutions have been prepared to have a pH of around 2 and 4, water has a pH of around 8 and basic solutions have a pH of around 10 and 13. The pH values are measured first using Hanna Instruments HI3222 pH meter. Line traces are made using manual screen printing of carbon ink that have an initial resistance, *R*_0_. The traces are exposed to 5 μl of each of the pH solutions. The resistance changed immediately after exposure and takes some time to stabilize due to absorption of the solution. The resistance is measured and the results are shown in Fig. 6(a). It can be seen that the resistance decreases with increasing pH values and different acid and base solutions with same pH produce similar change in resistance. The sensor only responds to the concentrations of H^ + ^and OH^−^ ions and the reading is not affected by the presence of other anions and cations.

Moreover, in order to confirm that the change in resistance is due to different pH values of the solutions and not due to their different conductivities, the conductivities of the solutions have also been measured and are shown in Fig. 6(b). The conductivities of pH 2 and pH 13 solutions are significantly higher which is due to the fact that these solutions have higher ionic concentrations. It can be seen that the trend of conductivity is different from that of resistance against different pH values. Hence, the resistance of the sensor does not seem to change due to conductivities of the solutions. The change in the resistance of the carbon based electrode can be due to the adsorption of OH^−^ and H^+^ ions on the electrode surface. Carbon in this case is acting like a conductor with free electrons as the charge carriers. The adsorption of H^+^ ions in the case of an acid result in the decrease in concentration of electrons on the surface of the electrode (H^+^ being an electron acceptor). This causes a decrease in conductivity. The effect of OH^−^ ions in the case of base is opposite, which result in the increased concentration of electron carriers and hence the conductivity. Similar behavior has been observed in the case of graphene^22^,^23^,^24^,^25^,^26^,^27^,^28^,^29^,^30^,^31^,^32^,^33^,^34^ and carbon nanotubes^35^. The maximum variation in the resistance change due to pH is around ±2.6%. The pH values in chronic wounds vary between 5.4 to 8.6 according to one study^36^. In this range of pH, the variation in measurements is small.

### System Tests

**Figure 7:**
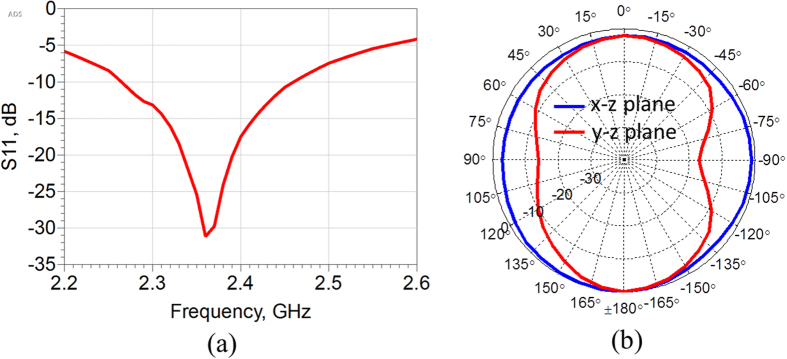
Antenna measurements. (**a**) Reflection coefficient (**b**) active radiation patterns.

The system level measurements have been performed that involve the wireless tests of the bandage with electronics mounted. Firstly an active radiation pattern measurement of the antenna has been measured. This is necessary as the antenna is surrounded by sensor electronics that can affect its radiation properties. The active pattern is shown in Fig. 7 along with the reflection coefficient of the antenna. As can be seen the active pattern is omnidirectional as expected from a loop antenna. Also the antenna is impedance matched at the operating frequency of 2.4 GHz.

**Figure 8:**
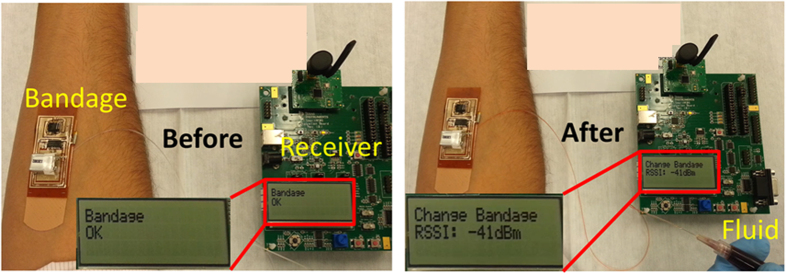
On-body test setup. The bandage (with exposed electronics) is worn on the forearm. When there is no bleeding, the receiver displays ‘Bandage OK’ sign as shown in the left picture. When there is bleeding, which is modeled by injecting blood mimicking fluid underneath the bandage, the bandage communicates with the receiver that displays ‘Change Bandage’ sign as shown in the right picture.

**Figure 9:**
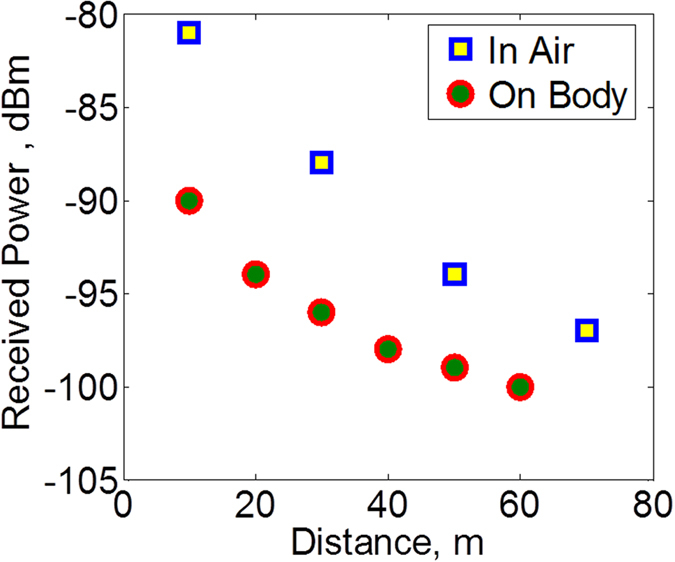
Range tests. The bandage can communicate to a receiver 60 m away when worn on the body. The deterioration in communication range is visible when the bandage is worn on the body as compared to when the bandage is in air due to the effects of body on the antenna performance.

The wireless tests are performed while the bandage is worn on the body. To carry out the tests, a fluid is injected underneath the bandage using a narrow tube that is attached to a syringe containing the fluid. The setup is shown in Fig. 8. This setup is made to imitate bleeding from the wound. A Zigbee wireless receiver is placed to receive an information signal from the bandage. When the fluid is pumped from the syringe and reaches the bottom side of the bandage, the transmitter on the bandage is activated and sends information to the receiver. Field tests have been carried out to measure the communication range of the bandage. The results are shown in Fig. 9. The range has been measured with the bandage on a human body as well as in air. As can be seen from the results, a lower range of around 60 m is observed when the bandage is placed on a body as the proximity effects of the body change the radiation characteristics of the antenna.

### Bending Response and Wearability tests

**Figure 10:**
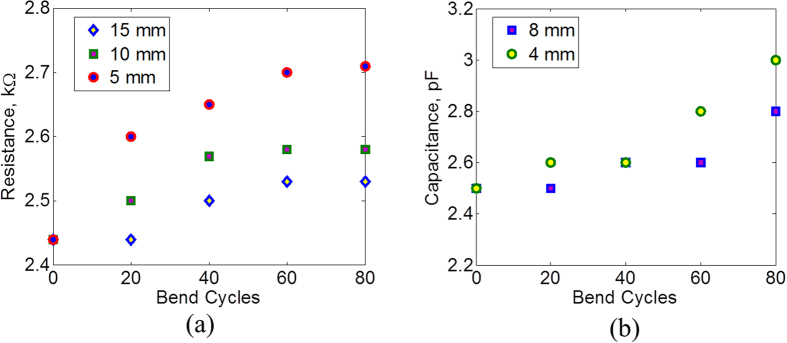
Effects of bending on sensor characteristics. (**a**) Resistance of the resistive sensor and (**b**) Capacitance of the capacitive sensor after bending cycles of different radii.

**Figure 11:**
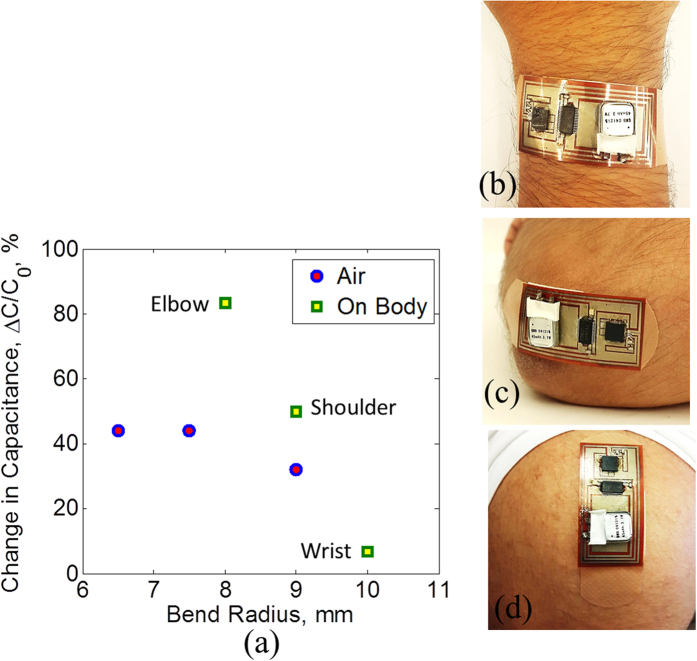
Wearablility tests. (**a**) Capacitance change under different bending radii and on body locations. Smart bandage (with exposed electronics) worn on (**b**) wrist (**c**) elbow and (**d**) shoulder.

Patients will wear the designed bandage, so it is necessary to evaluate the bandage performance in terms of flexibility and wearability. To perform flexibility tests, the bandage is subjected to several cycles of bending with different bending radii. The resistance of the bottom electrode and the capacitance across the bandage are measured after each bend cycle. Figure 10a,b show the results. The resistance varies only slightly after the bending cycles. The capacitance value also does not change much with maximum variation of about 0.5 pF. The capacitance of the sensor is also evaluated for different bending radii and also when worn on different body parts. The results are shown in Fig. 11. The capacitance changes when the bandage is bent in air. A change in capacitance is also observed when the bandage is worn on the body. This can be due to the proximity effect of the body that can change the fringing field of the capacitive sensor. The bandage is placed on the wrist, elbow and shoulder. The change in capacitance when worn on the wrist is minimal. The elbows and shoulders result in a bigger capacitance change due to the curvature of these joints. It should be mentioned that the wounds are typically located on body areas such as calves, foot surface and sacrum where bending is not likely to affect the performance of the bandage. Also, once worn on the body, the smart bandage has the capability to record the initial reading of the sensors which can be taken as the base reading. Any variation in the capacitance above this base reading will most likely be due to the bleeding or external pressure on typical wound locations. If the wounds are located on the joints like elbows and knees, bending can also cause the capacitance to change. Bleeding can be distinguished from bending based on the Q readings, Fig. 5(c), as bending is also expected to have less effect on the Q similar to pressure. Moreover, in the case of pressure ulcers, long term data of external pressure is important and few errors due to bending would have less effect on the overall outcome of the data. However, in order to reduce the errors due to bending on joints, separate sensors can be used for detecting bending and pressure (see Supplementary Fig. S2).

## Methods

### Fabrication

The bottom sensor electrode is made using carbon based ink manufactured by Bare Conductive. The ink is compatible with screen printing process. The ink is spread on office paper using a squeegee in similar fashion to screen printing. The sheet resistance of the trace formed by the ink is around 90 Ω/square. A laser is used to cut the paper along the pattern of the electrode. The electrode is then attached on the bottom side of the bandage using glue. A portion of the electrode is folded across the bandage so that it can be accessed from the top by the sensor electronics.

The sensor electronics has been fabricated on Kapton tape as the substrate which has a thickness of 110 μm and width of 1 in. A double-sided printed circuit board has been fabricated on the tape using inkjet printing. Silver nanoparticle based ink is used with an average particle size of 10 nm (UT Dots Inc. UTDAgIJ1). The inkjet printing is done using a Dimatix 2831 materials printer. Before printing, a laser is used to selectively remove the adhesive from the backside of the tape so that it becomes suitable for inkjet printing of the bottom side of the PCB. Also via holes are made using the laser to connect the two sides of the circuit. At this instance, the tape is cleaned with acetone to remove any contaminants. The circuit is inkjet printed with 80 um of drop spacing and 6 layers of ink. The tape is then put in the oven for sintering at 170 °C for 1 hour. The sheet resistance of the printed tracks on kapton tape is around 0.4 Ω/square. After printing the circuit, the components are mounted using silver epoxy paste, as typical soldering is not feasible for inkjet printed tracks due to the high temperature of the soldering process which is around 280 °C. The circuit is then attached to the bandage like a sticker in order to realize the complete system.

### On body experiments

The bandage was worn by an adult human to assess the wearability as well as the performance of the bandage under bent condition at different body locations. The communication range was measured using Texas Instruments SmartRF05 evaluation board and the resistance and capacitance was measured using Agilent E4982A LCR meter while the bandage was *in-situ*. Informed consent was obtained from the human subject prior to the experiments. All experiments were performed in accordance with applicable guidelines and regulations, and the experimental protocol for on body testing was approved by KAUST Institutional Biosafety and Bioethics Committee (Approval #15IBEC34).

## Discussion

A new low cost smart bandage system that can sense multiple parameters for chronic wound monitoring is presented in this work. The bandage can sense bleeding, pH levels and external pressure levels on the wound site providing important information for chronic wound treatment. The bandage can communicate wirelessly to inform remote medical staff about the status of the wound as well as inform the patient about the need to change the bandage. Measurements have shown that the bandage is suitable for wearability by a patient and requires low cost materials, and cheap and simple fabrication process. The proposed system provides an attractive platform for integration of additional sensors for wound monitoring. These can include temperature sensor and humidity sensor as both of these parameters play an important role in the wound healing process. This work can be further advanced by performing the *in-vivo* tests to establish the reliability of the system in real scenario. Moreover for real life application, a suitable packaging is required for detachable part of the smart bandage. One solution is to package the detachable electronics in a disposable and removable sleeve for the comfort of the patient as well as for the protection of reusable electronics. This will also eliminate any chances of contamination. Once used by a patient, the sleeve is replaced before the bandage is worn again by a patient. Similar design concept is used for modern clinical thermometers for covering their tips. Mobile applications can also be linked to the smart bandage system and can provide the patient with hand –held capability to monitor the wound healing process. For the case of pressure ulcer, the external pressure on the wound site can be monitored by a patient’s smart phone. The initial value of the sensor capacitance after wearing the bandage can be measured by the CDC and sent wirelessly to a patient’s smart phone or to a monitoring station. In case an external pressure is applied on the wound, the resulting change in capacitance is also communicated. A smart phone application can then compute the change in capacitance with respect to the initial value and relate this change to the pressure levels based on the results shown in Fig. 5b. The smart phone can then intimate the patient for repositioning in order to relieve the pressure. Going forward, this work can prove to be an important milestone in providing low cost health care services to an ever increasing global population, and thus reduces the burden on modern health care providers and government agencies.

## Additional Information

**How to cite this article**: Farooqui, M. F. and Shamim, A. Low Cost Inkjet Printed Smart Bandage for Wireless Monitoring of Chronic Wounds. *Sci. Rep.* **6**, 28949; doi: 10.1038/srep28949 (2016).

## Supplementary Material

Supplementary Information

## References

[b1] Honda W., Harada S., Arie T., Akita S. & Takei K. Wearable, human-interactive, health-monitoring wireless device fabricated by macroscale printing techniques. Adv. Funct. Mater. 24, 3299–3304 (2014).

[b2] Baig M. M., Gholamhosseini H. & Connolly M. J. A comprehensive survey of wearable and wireless ECG monitoring systems for older adults. Med. Biol. Engg. Comput . 51, 485–495 (2013). 10.1007/s11517-012-1021-6 23334714

[b3] Winokur E. S., Delano M. K. & Sodini C. G. A wearable cardiac monitor for long- term data acquisition and analysis. IEEE Trans. Biomedical Engg . 60, 189–192 (2013). 10.1109/TBME.2012.2217958 PMC4395508 22968205

[b4] Zheng Y.-L., Yan B. P., Zhang Y.-T. & Poon C. C. Y. An armband wearable device for overnight and cuff-less blood pressure measurement. IEEE Trans. Biomedical Engg . 61, 2179–2186 (2014). 10.1109/TBME.2014.2318779 24760899

[b5] Mihajlovic V., Grundlehner B., Vullers R. & Penders J. Wearable, wireless EEG solutions in daily life applications: What are we missing? IEEE J. Biomedical Health Informatics 19, 6–21 (2015). 10.1109/JBHI.2014.2328317 25486653

[b6] Sen C. K. . Human skin wounds: A major and snow balling threat to public health and economy. *Wound Repair Regen*. 17, 763–771 (2009). 19903300 10.1111/j.1524-475X.2009.00543.x PMC2810192

[b7] Posnett J., Gottrup F., Lundgren H. & Saal G. The resource impact of wounds on health-care providers in Europe. J. Wound Care 18, 154–161 (2009). 19349935 10.12968/jowc.2009.18.4.41607

[b8] Nunan R., Harding K. G. & Martin P. Clinical challenges of chronic wounds: searching for an optimal animal model to recapitulate their complexity. Disease Models & Mechanisms . 7, 1205–1213 (2014). 25359790 10.1242/dmm.016782 PMC4213725

[b9] Blakytny R. & Jude R. E. The molecular biology of chronic wounds and delayed healing in diabetes. Diabet. Med . 6, 594–608 (2006). 16759300 10.1111/j.1464-5491.2006.01773.x

[b10] Gist S., Matos I. T., Falzgraf S., Cameron S. & Beebe M. Wound care in the geriatric client. Clin. Interv. Aging . 4, 269–287 (2009). 19554098 10.2147/cia.s4726 PMC2697592

[b11] Gardner S. E., Frantz R. A. & Doebbeling B. N. The validity of the clinical signs and symptoms used to identify localized chronic infection. Wound Repair and Regeneration. 9, 178–186 (2001). 11472613 10.1046/j.1524-475x.2001.00178.x

[b12] Hampton S. Understanding overgranulation in tissue viability practice. British Journal of Community Nursing. 12 (2007). 10.12968/bjcn.2007.12.Sup4.43000 18026011

[b13] Schreml S., Szeimies R.-M., Karrer S., Heinlin J., Landthaler M. & Babilas P. The impact of the pH value on skin integrity and cutaneous wound healing. J. European Academy Dermatology Venereology . 24, 373–378 (2010). 10.1111/j.1468-3083.2009.03413.x 19703098

[b14] Dharmarajan T. S. & Ugalino J. T. Pressure ulcers: Clinical features and management. Hospital Physician . 38, 64–71 (2002).

[b15] Li Z. . Non-invasive transdermal two-dimensional mapping of cutaneous oxygenation with a rapid drying liquid bandage. Biomedical Optics Express. 5, 3748–3764 (2014). 25426308 10.1364/BOE.5.003748 PMC4242015

[b16] Guinovart T., Ramirez G. V.-, Windmiller J. R., Andrade F. J. & Wang J. Bandage-based wearable potentiometric sensor for monitoring wound pH. Electroanalysis. 26, 1345–1353 (2014).

[b17] Swisher S. L. . Impedance sensing device enables early detection of pressure ulcers *in vivo*. Nature Commun . 6, 6575 (2015). 25779688 10.1038/ncomms7575

[b18] Farrow M. J., Hunter I. S. & Connolly P. Developing a real time sensing system to monitor bacteria in wound dressings. *Biosensors*. 2, 171–188 (2012). 25585709 10.3390/bios2020171 PMC4263571

[b19] McColl D., Cartlidge B. & Connolly P. Real-time monitoring of moisture levels in wound dressings *in vitro*: An experimental study, International J. Surgery . 5, 316–322 (2007). 10.1016/j.ijsu.2007.02.008 17499032

[b20] Sridhar V. & Takahata K. A hydrogel-based passive wireless sensor using a flex-circuit inductive transducer. Sensors Actuators A . 155, 58–65 (2009).

[b21] Mehmood N., Hariz A., Templeton S. & Voelcker N. H. An improved flexible telemetry system to autonomously monitor sub-bandage pressure and wound moisture. *Sensors*. 14, 21770–2170 (2014). 25412216 10.3390/s141121770 PMC4279561

[b22] Steckl A. J. Circuits on cellulose. IEEE Spectrum. 50, 48–61 (2013).

[b23] Cook H. F. A comparison of the dielectric behavior of pure water and human blood at microwave frequencies. British Journal of Applied Physics. 3 (1952).

[b24] Andreuccetti D., Fossi R. & Petrucci C. Dielectric properties of body tissues . Available at: http://niremf.ifac.cnr.it/tissprop/htmlclie/htmlclie.php. (Accessed: 21^st^ May 2016).

[b25] Gabriel S., Lau R. W. & Gabriel C. The dielectric properties of biological tissues: III. Parametric models for the dielectric spectrum of tissues. Physics in Medicine and Biology 41, 2271 (1996). 8938026 10.1088/0031-9155/41/11/003

[b26] Cutting K. Wound exudate: composition and functions. Br. J. Community Nurs . 8, 4–9 (2003). 14685963 10.12968/bjcn.2003.8.sup3.11577

[b27] Harvey C. J., LeBouf R. F. & Stefaniak A. B. Formulation and stability of a novel artificial human sweat under conditions of storage and use. Toxicology in Vitro. 24, 1790–1796 (2010). 20599493 10.1016/j.tiv.2010.06.016

[b28] Romanov A. N. Dielectric properties of human sweat fluid in the microwave range. Biophysics 55, 473–476 (2010). 20586336

[b29] Patterson J. A. & Bennett R. G. Prevention and treatment of pressure sores. J Am. Geriatr. Soc. 43, 919–927 (1995). 7636103 10.1111/j.1532-5415.1995.tb05538.x

[b30] Witkowski J. A. & Parish L. C. Histopathology of the decubitus ulcer. J. Am. Acad. Dermatol. 6, 1014–1021 (1982). 7096663 10.1016/s0190-9622(82)70085-4

[b31] Analog Devices. 1 MSPS, 12 bit impedance converter network analyzer. Available at: http://www.analog.com/media/en/technical-documentation/data-sheets/AD5933.pdf. (Accessed: 21^st^ May 2016).

[b32] Lei N., Li P., Xue W. & Xu J. Simple graphene chemiresistors as pH sensors: fabrication and characterization. Meas. Sci. Technol. 22, 107002 (2011).

[b33] Ang P. K., Chen W., Wee A. T. S. & Loh K. P. Solution-gated epitaxial graphene as pH sensor. J. Am. Chem. Soc. 130, 14392–14393 (2008). 18850701 10.1021/ja805090z

[b34] Ohno Y., Maehashi K., Yamashiro Y. & Matsumoto K. Electrolyte-gated graphene field-effect transistors for detecting pH and protein adsorption. Nano Lett. 9, 3318–3322 (2009). 19637913 10.1021/nl901596m

[b35] Jung D., Han M.-E. & Lee G. S. pH-sensing characteristics of multi-walled carbon nanotube sheet. Materials Letters. 116, 57–60 (2014).

[b36] Percival S. L., McCarty S., Hunt J. A. & Woods E. J. The effects of pH on wound healing, biofilms,and antimicrobial efficacy. Wound Rep. Reg . 22, 174–186 (2014). 10.1111/wrr.12125 24611980

