# Supplementary material for: Low Cost Inkjet Printed Smart Bandage for Wireless Monitoring of Chronic Wounds
Source: Sci Rep. 2016 Jun 29;6:28949. doi: 10.1038/srep28949 (PMC4926082; doi:10.1038/srep28949)
Supplement: Supplementary Information [file srep28949-s1.doc]

Low Cost Inkjet Printed Smart Bandage for Wireless Monitoring of Chronic Wounds

Muhammad Fahad Farooqui^a^ and Atif Shamim^a^,*

^a^ Electrical Engineering Program, 4700 King Abdullah University of Science and Technology (KAUST), Thuwal 23955-6900, Saudi Arabia
*Corresponding author. E-mail: atif.shamim@kaust.edu.sa


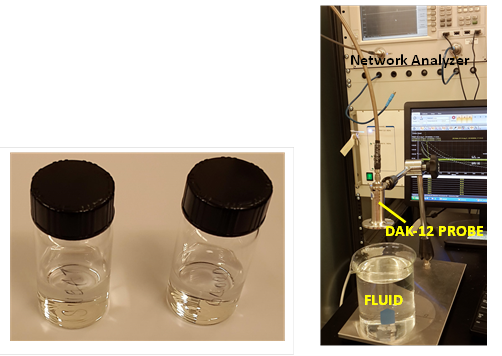


**Supplementary Figure S1. Mimicking fluids for blood and sweat.** (a) Blood has been modeled by a mixture of ethanol and saline solution, and sweat has been modeled by a saline solution (b) Measurement setup for measuring the dielectric properties of fluids. Measured properties, blood: ε_r_= 63, tan δ= 1.24, sweat: ε_r_= 79, tan δ= 1.05


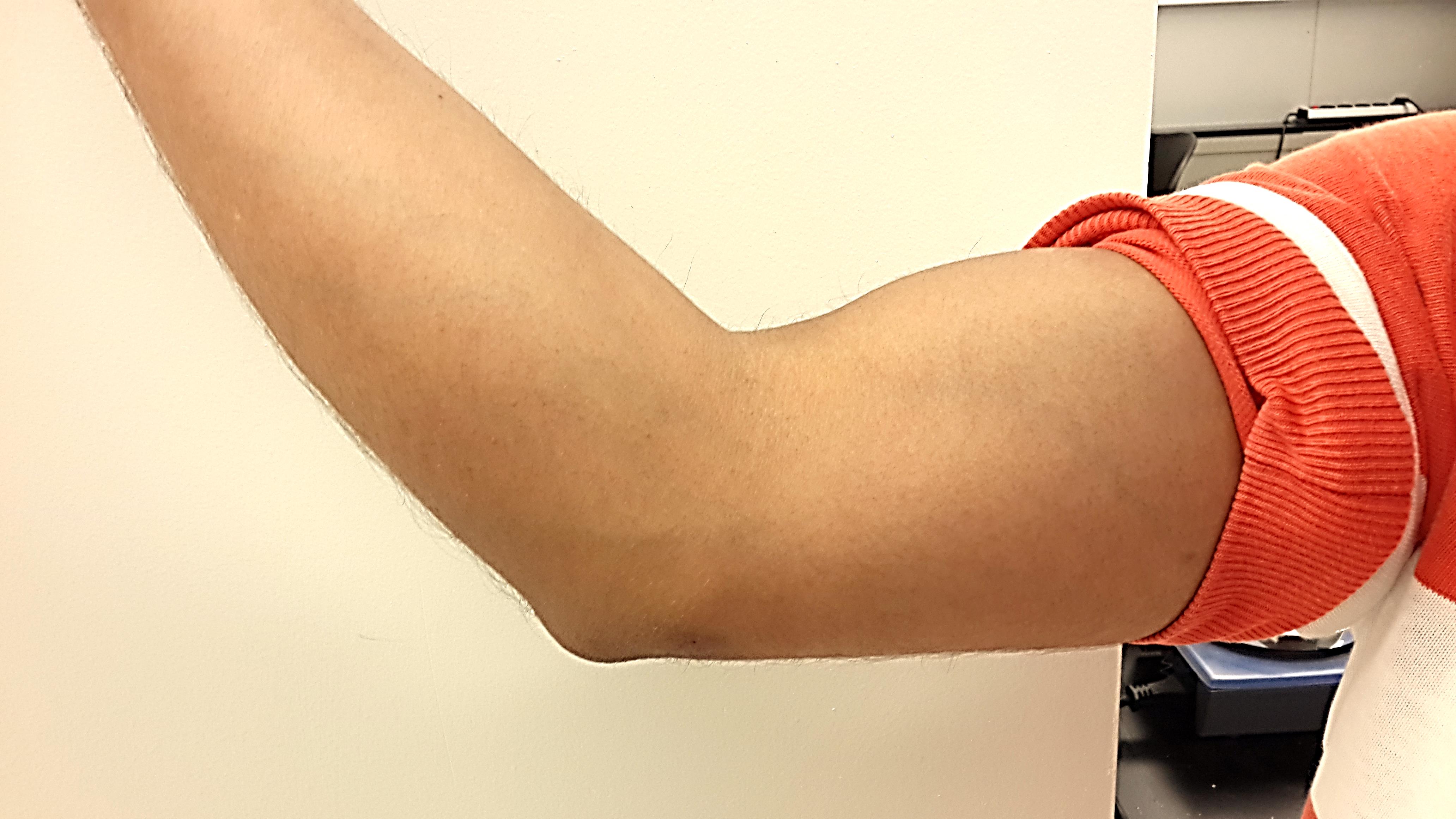

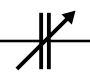

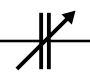


**Outer elbow**

**Sensor for BENDING+PRESSURE**

**Inner elbow**

**Sensor for BENDING**

**Bed or Chair**

**BENDING**


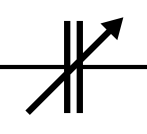

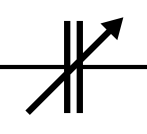


**OP AMP**

**BENDING+PRESSURE**

**Supplementary Figure S2. Differential measurements on the joints (elbow).** (a) Placement of separate sensors for bending and pressure. (b) Schematic of the circuit for differential measurements using operational amplifier (op-amp) inside the CDC.
